# Incidence and prevalence of systemic lupus erythematosus in New Zealand from the national administrative datasets

**DOI:** 10.1177/09612033231182203

**Published:** 2023-06-02

**Authors:** Chunhuan Lao, Douglas White, Kannaiyan Rabindranath, Philippa Van Dantzig, Donna Foxall, Apo Aporosa, Ross Lawrenson

**Affiliations:** 1 Medical Research Centre, 3717 The University of Waikato, Hamilton, New Zealand; 2 Rheumatology Department, 3718 Waikato Hospital, Hamilton, New Zealand; 3 Renal Unit, 3718 Waikato Hospital, Hamilton, New Zealand; 4 Te Huataki Waiora - School of Health, 3717 The University of Waikato, Hamilton, New Zealand; 5 School of Psychology, 3717 The University of Waikato, Hamilton, New Zealand; 6 Strategy and Funding, 3718 Waikato Hospital, Hamilton, New Zealand

**Keywords:** Systemic lupus erythematosus, lupus, incidence, prevalence, ethnic difference

## Abstract

**Objectives:**

This study aims to provide updated data on the incidence and prevalence of systemic lupus erythematosus (SLE) in New Zealand and to examine the difference between ethnic groups.

**Methods:**

We identified the SLE cases from the national administrative datasets. The date of first identification of SLE was the earliest date of a related inpatient event or the earliest date of a related outpatient event. The crude incidence and prevalence of SLE in 2010–2021 were estimated by gender, age group and ethnicity. The WHO (World Health Organization) age-standardised rate (ASR) of incidence and prevalence of SLE was calculated, after stratifying the cases by ethnicity and gender.

**Results:**

The average ASR of incidence and prevalence of SLE in 2010–2021 was 2.1 and 42.1 per 100,000 people in New Zealand. The average ASR of incidence for women was 3.4 per 100,000 for women and 0.6 for men. It was highest for Pacific women (9.8), followed by Asian women (5.3) and Māori women (3.6), and was lowest for Europeans/Others (2.1). The average ASR of prevalence was 65.2 per 100,000 for women and 8.5 for men. It was highest for Pacific women (176.2), followed by Māori women (83.7) and Asian women (72.2), and was lowest for Europeans/Others (48.5). The ASR of prevalence of SLE has been increasing slightly over time: from 60.2 in 2010 to 66.1 per 100,000 in 2021 for women and from 7.6 in 2010 to 8.8 per 100,000 in 2021 for men.

**Conclusion:**

The incidence and prevalence of SLE in New Zealand were comparable to the rates in European countries. Pacific people had the highest incidence and prevalence of SLE, more than three times the rates for Europeans/others. The high incidence of SLE in Māori and Asian people also has implications for the future as these populations increase as a proportion to the total population.

## Introduction

Systemic lupus erythematosus (SLE) is the prototypic autoimmune disease, where the body’s immune system attacks its own tissue and organs.^1^ Inflammation caused by SLE can affect many different body systems, including joints, skin, central nervous system, lungs, kidneys, gastro-intestinal tract, cardiovascular system and bone marrow.^1^ Patients with SLE may suffer from severe fatigue, joint pain, headaches, malar or ‘butterfly rash’, alopecia, anaemia, thrombosis, Raynaud’s phenomenon and other symptoms depending on the part of the body the disease is attacking. Its natural history is characterised by episodes of relapses or flares interchanging with remissions, and the outcome is highly variable, ranging from permanent remission to death.^2^

SLE is the commonest type of lupus, with about 70% of the lupus cases being systemic. It was reported that SLE affects millions of people worldwide.^3^ The global incidence and prevalence of SLE vary widely in different countries.^4^ The incidence of SLE was reported to be between 3.7 and 49.0 per 100,000 person-years in the US Medicare population in North America, 1.5 and 7.4 in Europe, 1.4 and 6.3 in South America and 2.5 and 8.6 in Asia. Estimates of the current incidence of SLE in Australasia or Africa are unavailable. The prevalence of SLE varies between 48 and 366.6 per 100,000 person-years in North America, 29.3 and 210 in Europe, 24.3 and 126.3 in South America, 20.6 and 103 in Asia, 13 and 52 in Australasia and 601.3 and 7713.5 in Africa.^4^

The epidemiology of SLE varies by age, gender and ethnicity. Most SLE patients are women, and the reported sex ratio ranged from 2:1 to 15:1 for women compared to men.^5^ Most of the SLE cases were between the ages of 15 and 45 years.^6^ The prevalence differs by ethnicity – SLE is more common and of greater severity in Australian Aboriginal, Asian, Polynesian, African Americans and Hispanic people.^7,8^ For example, the prevalence of SLE in the United States was reported to be 230.9 per 100,000 for African women, 120.7 for Hispanic women, compared to 84.7 for European descendants and 84.4 for Asian/Pacific women.^8^ The prevalence for American men by ethnicity followed the same pattern.^8^ In New Zealand, the incidence of juvenile SLE has been reported as being higher in Māori compared with European children and Māori children are more likely to be diagnosed with lupus nephritis.^9^

New Zealand is a multicultural country. The majority of New Zealand’s five million population are New Zealand Europeans (70%), followed by the indigenous Māori people (16%), then Asian (15%) and then Pacific people (8%). There are very few studies available examining the epidemiology of SLE in New Zealand.^10–14^ The only paper showing the prevalence of SLE in New Zealand was published in 1983, demonstrating the age-adjusted prevalence rates of 14.6 per 100,000 people for NZ Europeans, 50.6 for Polynesian and 19.1 for others.^14^ This study aims to provide updated data on the incidence and prevalence of SLE in New Zealand and to examine the difference between ethnic groups, using the national administrative datasets.

## Methods

We first identified the SLE cases between 1 January 2005 and 31 December 2021 using the ICD-10 code ‘M32’ from the National Minimum Dataset (NMDS) and the Mortality Collection (coded death records) and using the key words ‘systemic lupus erythematosus’ from the Death Certificates (uncoded death records). The first date in the NMDS for an inpatient event with an ICD-10 code of ‘M32’ or the first date from the National Non-admitted Patient Collection (NNAPC) for an outpatient event in the Rheumatology department or Renal Service, whichever date was earlier was considered as the date of first identification of SLE. The NMDS records inpatient and day patient discharges from all public hospitals and over 90% of private hospitals. The NNAPC includes event-based purchase units that relate to medical and surgical outpatient events and emergency department events from both public and private hospitals. Mortality Collection and Death Certificates contains information about all deaths registered in New Zealand, and the Death Certificates collects more up-to-date death records not included in the Mortality Collection yet. The NMDS and the NNAPC start collecting data from 2005, and the NNAPC does not include ICD codes. These datasets were linked together through their National Health Index (NHI) number. NHI is a unique identifier assigned to every person who uses health and disability Services in New Zealand. It can accurately identify people and link them with the right health records.

To validate our methods for identifying SLE patients and date of SLE identification using the national administrative datasets, we compared the data from national administrative datasets with the medical records using the Waikato patients. For these patients in the Waikato region, we examined their medical records in the Clinical Workstation in Waikato hospital to confirm the diagnosis of SLE and to identify the first date of SLE identification. The date of SLE identification in the clinical records was compared with the date from the national administrative datasets. The gap between these two dates was categorised into 0 year, 1–5 years, 6–10 years, and no lupus information in the Waikato hospital. Patients who immigrated from other regions to the Waikato region in recent years may not have outpatient records in the Rheumatology department or Renal Service nor inpatient records in the Waikato hospital. The earliest date of SLE identification was used for following data analysis if the two dates were different.

To reduce the bias on date of SLE identification in the national administrative datasets since these datasets only started collecting data from 2005, we only estimated the incidence and prevalence of SLE in 2010–2021. The incidence was based on the 2010–2021 data and the prevalence was based on the 2005–2021 data. Patient characteristics of the SLE patients in 2010–2021 were described and compared by ethnic group (Māori, Pacific, Asian and Europeans/Others) used in the 2018 New Zealand Census.^15^ Ethnicity is self-identified in New Zealand. The Asian group included Chinese, Japanese, Korean, Southeast Asian, Indian, Sri Lankan and other Asian (not further defined).^15^ Europeans included New Zealand European, British and Irish, Italian, German, South Slav, Scandinavian, South African European and Europeans not elsewhere classified.^15^ Characteristics examined included gender (women and men), age (<20, 20–29, 30–39, 40–49, 50–59, 60–69, 70–79 and 80+ years), Charlson Comorbidity Index (CCI) score, and socioeconomic deprivation. Socioeconomic deprivation was defined using the New Zealand Index of Deprivation 2018 (NZDep 2018) analysed as quintile, from 1 (least deprived) to 5 (most deprived).^16^ Comorbidities recorded in the NMDS before or on the date of SLE identification were included in the CCI score calculation. Differences in patient characteristics by ethnic group were examined with Chi-square test, with a *p*-value of less than 0.05 considered significant.

The crude incidence and prevalence of SLE were estimated by age group after stratifying the patients by gender. For incidence in different years, the age at first identification was used for the calculation, while for prevalence, the age in the prevalence year was used. The population data used for calculating the incidence and prevalence rates were based on the 2006, 2013 and 2018 Census data.^17^ The population in the years when the Census data were not available were estimated assuming that the population grow steadily using the Census populations. The WHO (World Health Organization) age-standardised rate (ASR) of incidence and prevalence of SLE and 95% confidence interval (CI) was calculated, after stratifying the cases by ethnicity and gender. All data analyses were performed in R 4.0 (R Institute, Vienna, Austria). Ethics approval for the study was granted through the Northern B Health and Disability Ethics Committee (reference: 2022 EXP 13741).

## Results

We have identified 2837 SLE patients in 2005–2021 from the national administrative datasets, including 1657 first identified in 2005–2009, 1145 in 2010–2021 and 35 with unknown date of first identification. For patients identified in 2005–2009, some were prevalent cases (diagnosed before 2005), and patients in 2010–2021 were the newer/incident cases. This is because the administrative datasets collect data from 2005. Of these 2837 patients, 433 (15.3%) were Māori, 433 (15.3%) Pacific, 420 (14.8%) Asian and 1551 (54.7%) Europeans/others. After examining the clinical records of the 200 patients in the Waikato region, we found that the gap between the date of first SLE identification in the Waikato hospital records and the date from the national administrative datasets was small for patients in 2010–2021 (Appendix Table 1). For patients identified in 2010–2021 from the national administrative datasets, 78.4% of patients had no gap between the two dates and 11.4% had a gap of 1–5 years, compared to 45.5% of patients having no gap and 24.8% having a gap of 1–5 years between the two dates for patients first identified in 2005–2009.

The number of SLE patients identified per year was generally stable in 2010–2021 (Table 1). Of the 1145 patients in 2010–2021, 960 (83.8%) were women and 185 (16.2%) were men. Patients identified at the age of 30–39 years accounted for the largest proportion, followed by patients at the age of 20–29 years. Nearly 80% of the patients were identified under the age of 60 years old. This figure was highest for Pacific people (91.6%), followed by Asian (91.1%) and Māori (88.0%), and was lowest for European/Others (63.9%).

**Table 1.** Characteristics of patients identified in 2010–2021 from the national administrative datasets.

Subgroup	Māori		Pacific		Asian		European/Others			Total	
Year of first identification											
2010	23	(13.1%)	24	(11.2%)	18	(7.7%)	67	(12.9%)	<0.001	132	(11.5%)
2011	25	(14.3%)	21	(9.8%)	15	(6.4%)	50	(9.6%)		111	(9.7%)
2012	16	(9.1%)	17	(7.9%)	13	(5.5%)	61	(11.7%)		107	(9.3%)
2013	10	(5.7%)	16	(7.5%)	16	(6.8%)	49	(9.4%)		91	(7.9%)
2014	11	(6.3%)	15	(7.0%)	31	(13.2%)	38	(7.3%)		95	(8.3%)
2015	9	(5.1%)	25	(11.7%)	24	(10.2%)	47	(9.0%)		105	(9.2%)
2016	16	(9.1%)	17	(7.9%)	26	(11.1%)	44	(8.4%)		103	(9.0%)
2017	13	(7.4%)	15	(7.0%)	23	(9.8%)	37	(7.1%)		88	(7.7%)
2018	8	(4.6%)	17	(7.9%)	15	(6.4%)	31	(6.0%)		71	(6.2%)
2019	13	(7.4%)	16	(7.5%)	19	(8.1%)	40	(7.7%)		88	(7.7%)
2020	16	(9.1%)	9	(4.2%)	18	(7.7%)	32	(6.1%)		75	(6.6%)
2021	15	(8.6%)	22	(10.3%)	17	(7.2%)	25	(4.8%)		79	(6.9%)
Gender											
Female	144	(82.3%)	190	(88.8%)	205	(87.2%)	421	(80.8%)	0.021	960	(83.8%)
Male	31	(17.7%)	24	(11.2%)	30	(12.8%)	100	(19.2%)		185	(16.2%)
Age group (years)											
<20	36	(20.6%)	53	(24.8%)	47	(20.0%)	38	(7.3%)	<0.001	174	(15.2%)
20–29	39	(22.3%)	57	(26.6%)	44	(18.7%)	65	(12.5%)		205	(17.9%)
30–39	26	(14.9%)	46	(21.5%)	67	(28.5%)	72	(13.8%)		211	(18.4%)
40–49	28	(16.0%)	27	(12.6%)	32	(13.6%)	79	(15.2%)		166	(14.5%)
50–59	25	(14.3%)	13	(6.1%)	24	(10.2%)	79	(15.2%)		141	(12.3%)
60–69	11	(6.3%)	13	(6.1%)	15	(6.4%)	91	(17.5%)		130	(11.4%)
70–79	10	(5.7%)	5	(2.3%)	3	(1.3%)	55	(10.6%)		73	(6.4%)
80+	0	(0.0%)	0	(0.0%)	3	(1.3%)	42	(8.1%)		45	(3.9%)
Deprivation quintile											
1 (least deprived)	6	(3.4%)	11	(5.4%)	45	(19.3%)	98	(19.0%)	<0.001	160	(14.2%)
2	17	(9.8%)	10	(4.9%)	47	(20.2%)	105	(20.3%)		179	(15.9%)
3	22	(12.6%)	20	(9.8%)	41	(17.6%)	119	(23.1%)		202	(17.9%)
4	38	(21.8%)	48	(23.4%)	58	(24.9%)	119	(23.1%)		263	(23.3%)
5 (most deprived)	91	(52.3%)	116	(56.6%)	42	(18.0%)	75	(14.5%)		324	(28.7%)
Unknown	1		9		2		5			17	
CCI score											
0	135	(77.1%)	156	(72.9%)	207	(88.1%)	352	(67.6%)	<0.001	850	(74.2%)
1	24	(13.7%)	40	(18.7%)	17	(7.2%)	96	(18.4%)		177	(15.5%)
2	5	(2.9%)	13	(6.1%)	9	(3.8%)	35	(6.7%)		62	(5.4%)
3	7	(4.0%)	2	(0.9%)	1	(0.4%)	19	(3.6%)		29	(2.5%)
4+	4	(2.3%)	3	(1.4%)	1	(0.4%)	19	(3.6%)		27	(2.4%)
**Total**	**175**		**214**		**235**		**521**			**1145**	

**Figure 1.**
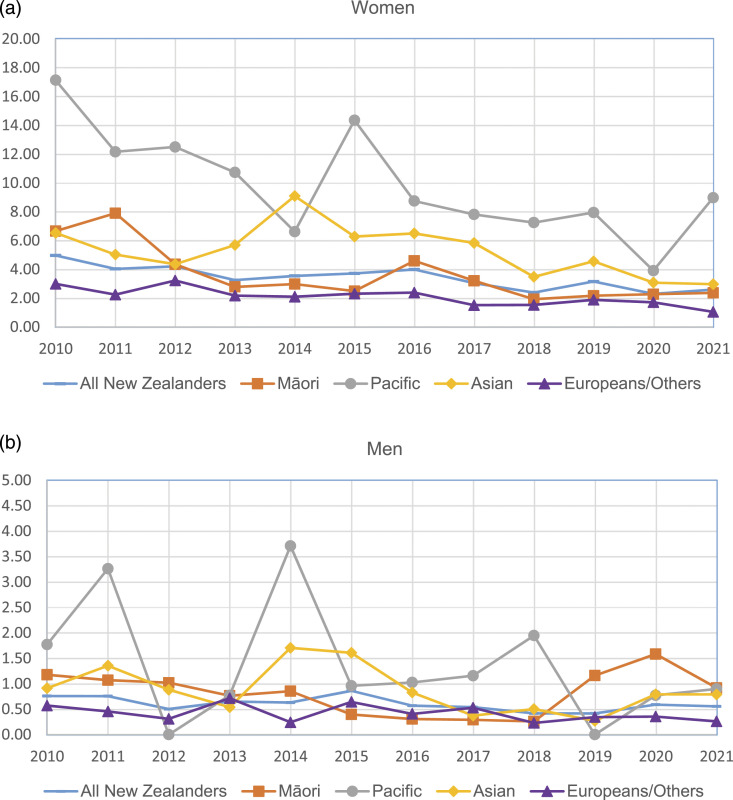
Age-standardised incidence rate of SLE by ethnic group by year: (a) women; (b) men.

The average crude incidence of SLE in New Zealand (Table 2) in 2010–2021 was 2.1 per 100,000 people and was 3.5 for women and 0.7 for men. For women, the incidence was lowest for those under 20 years old (2.2) and was highest for women aged 30–39 years (5.2) followed by women aged 20–29 years (4.9). The average ASR of incidence in 2010–2021 for women was 3.4 (95% CI: 2.7–4.2) per 100,000 for women (Figure 1, Appendix Table 2). It was highest for Pacific women (9.8, 95% CI: 5.1–14.6), followed by Asian women (5.3, 95% CI: 2.8–7.8) and Māori women (3.6, 95% CI: 1.6–5.6), and was lowest for Europeans/Others (2.1, 95% CI: 1.4–2.8).

**Table 2.** Crude incidence rate of SLE in New Zealand by age group (per 100,000 people).

Subgroup	2010	2011	2012	2013	2014	2015	2016	2017	2018	2019	2020	2021	Average
Women													
Age (years)													
0–19	2.9	2.6	2.3	1.4	1.7	2.0	2.2	1.8	1.7	3.3	1.3	2.6	2.2
20–29	4.6	3.1	5.9	5.8	6.0	6.8	5.4	5.5	4.8	4.1	3.1	3.6	4.9
30–39	6.1	7.6	8.7	3.9	5.5	5.4	7.9	4.8	1.9	4.3	4.2	2.4	5.2
40–49	7.5	4.4	2.8	4.6	4.3	3.1	5.3	2.2	1.6	2.2	2.5	2.5	3.6
50–59	6.8	5.2	2.7	3.0	3.3	3.9	3.2	1.9	2.5	2.2	1.2	1.8	3.1
60–69	7.7	5.1	5.8	2.6	2.1	4.1	2.4	2.0	2.3	2.2	2.2	2.5	3.4
70–79	4.6	1.5	4.4	2.9	4.1	2.0	0.6	4.9	2.3	1.7	2.7	1.6	2.8
80+	2.3	5.5	3.3	6.4	0.0	1.0	2.0	4.0	4.0	3.9	1.0	1.9	2.9
Overall	5.3	4.2	4.2	3.4	3.5	3.7	3.8	3.1	2.4	3.0	2.3	2.5	3.5
Men													
Age (years)													
0–19	0.2	0.2	0.3	0.3	0.5	0.7	0.2	0.2	0.0	0.3	0.3	0.3	0.3
20–29	0.7	0.0	0.4	0.4	0.3	0.3	0.9	0.9	0.9	0.0	0.5	1.0	0.5
30–39	1.1	1.9	0.0	0.4	0.4	1.8	1.0	0.3	0.3	0.3	0.0	0.3	0.7
40–49	1.7	1.4	0.3	2.4	0.3	0.7	0.7	1.0	0.3	0.3	0.3	0.0	0.8
50–59	1.1	0.7	1.1	0.0	1.4	0.7	0.7	1.0	0.3	0.0	1.6	0.6	0.8
60–69	1.0	1.0	1.4	0.9	1.8	1.7	0.0	0.4	1.2	0.8	2.3	1.5	1.2
70–79	0.8	2.5	1.6	0.8	1.5	0.7	0.7	0.7	1.9	2.5	1.2	0.6	1.3
80+	0.0	1.7	1.7	1.6	0.0	1.5	1.5	1.4	0.0	4.1	0.0	2.6	1.4
Overall	0.8	0.9	0.6	0.7	0.7	0.9	0.6	0.6	0.5	0.5	0.7	0.6	0.7
All people													
Overall	3.1	2.6	2.5	2.1	2.1	2.3	2.2	1.9	1.5	1.8	1.5	1.6	2.1

**Figure 2.**
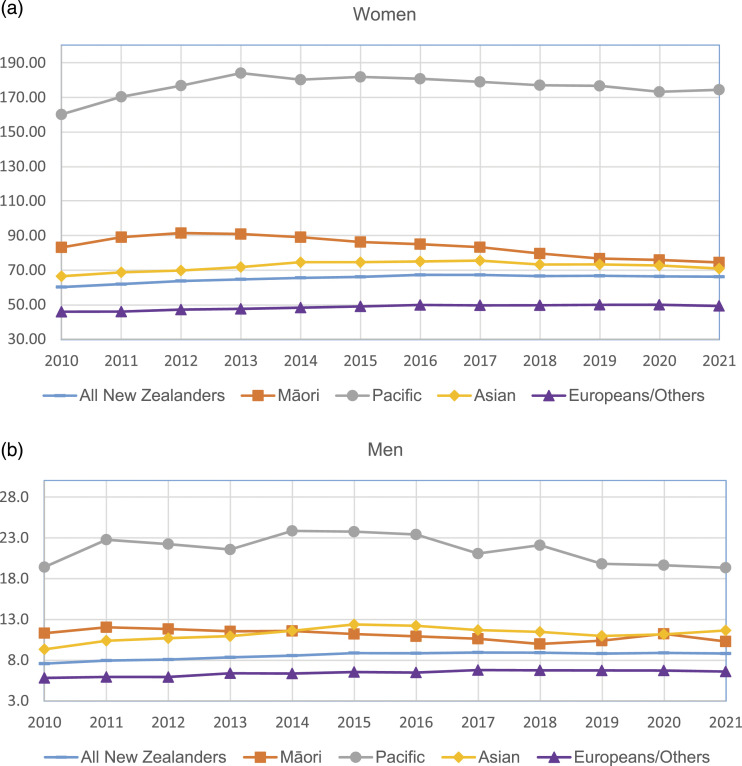
Age-standardised prevalence rate of SLE by ethnic group by year: (a) women; (b) men.

The average crude prevalence of SLE in New Zealand (Table 3) in 2010–2021 was 42.1 per 100,000 people, 73.1 for women and 9.6 for men. For women, the prevalence was lowest for those under 20 years old (11.0) and was highest for women aged 40–49 years (121.9) followed by women aged 50–59 years (103.6). The average ASR of prevalence in 2010–2021 was 65.2 (95% CI: 62.1–68.3) per 100,000 for women (Figure 2, Appendix Table 3). It was highest for Pacific women (176.2, 95% CI: 155.0–197.4), followed by Māori women (83.7, 95% CI: 73.8–93.6) and Asian women (72.2, 95% CI: 63.2–81.2), and was lowest for Europeans/Others (48.5, 95% CI: 45.3–51.6). The ASR of prevalence of SLE has been increasing slightly over time: from 60.2 in 2010 to 66.1 per 100,000 in 2021 for women and from 7.6 in 2010 to 8.8 in 2021 for men.

**Table 3.** Crude prevalence rate of SLE in New Zealand (per 100,000 people).

Subgroup	2010	2011	2012	2013	2014	2015	2016	2017	2018	2019	2020	2021	Average
Women													
Age (years)													
0–19	11.6	11.6	12.0	10.4	10.3	10.7	11.0	10.9	10.1	11.7	10.4	11.5	11.0
20–29	59.7	60.6	60.8	65.1	66.6	65.1	66.7	62.5	61.2	59.7	56.2	52.6	61.4
30–39	109.3	110.5	112.5	107.1	104.4	103.6	102.5	102.4	98.2	93.6	95.5	93.2	102.7
40–49	94.0	101.0	107.0	115.1	120.6	123.7	129.9	132.7	130.8	135.2	137.7	134.9	121.9
50–59	93.6	96.9	95.0	95.8	98.6	104.3	106.6	107.0	113.5	111.6	108.2	111.8	103.6
60–69	88.0	91.0	99.6	98.2	99.8	99.4	99.4	102.5	104.3	104.5	105.1	106.0	99.8
70–79	79.4	76.2	82.0	92.6	89.3	87.0	83.5	84.6	82.1	87.7	93.5	97.3	86.3
80+	37.1	43.2	39.2	45.0	50.6	46.7	54.2	59.4	61.5	60.6	60.7	60.8	51.6
Overall	66.1	68.2	70.3	71.9	73.2	73.9	75.3	75.7	75.5	75.8	75.6	75.6	73.1
Men													
Age (years)													
0–19	2.2	1.8	1.5	1.8	1.7	2.3	2.1	2.1	1.9	1.9	2.0	1.8	1.9
20–29	5.4	5.0	6.4	6.3	7.1	6.8	7.5	7.8	7.8	6.4	6.7	7.3	6.7
30–39	9.0	10.6	11.1	10.1	9.4	10.1	8.7	8.8	8.8	10.1	9.5	8.9	9.6
40–49	12.5	13.9	12.9	15.0	14.9	13.8	14.8	15.1	15.0	13.9	12.9	12.5	13.9
50–59	12.5	11.5	12.8	12.3	13.8	16.0	16.7	16.1	16.5	16.6	18.5	19.8	15.3
60–69	15.1	17.0	16.4	16.4	19.1	19.4	16.4	17.3	18.1	17.3	19.2	17.7	17.5
70–79	15.3	17.4	17.0	16.6	15.1	15.9	18.7	20.0	20.5	22.8	20.2	20.1	18.3
80+	17.8	20.6	19.9	25.6	24.9	18.2	17.8	14.5	12.7	15.2	13.5	17.2	18.2
Overall	8.7	9.2	9.4	9.7	10.1	10.3	10.3	10.4	10.4	10.4	10.6	10.6	10.0
All people													
Overall	38.1	39.5	40.6	41.6	42.4	42.8	43.4	43.6	43.4	43.5	43.4	43.3	42.1

## Discussion

This study provided updated data on both the incidence and prevalence of SLE in New Zealand and demonstrated the ethnic differences in SLE.^14^ The estimated incidence and prevalence in New Zealand were lower than the rates in North America but were comparable to the rates reported in some countries in Europe.^4^ The prevalence of SLE has increased substantially since the 1983 study.^14^ For example, the prevalence of SLE for Europeans increased from 14.6 per 100,000 in 1983 to 43.3 per 100,000 in 2021.^14^ The increasing trend in SLE prevalence has also been observed in overseas studies, while the incidence has been relatively stable over time.^18,19^ The biggest increase in prevalence was in the elderly. Our study found that patients aged 40–49 years had the highest prevalence which is consistent with other studies.^18^ The increasing SLE prevalence was probably because of the improving life expectancy and advancing treatments for SLE and lupus nephritis.^20^

Most of the SLE patients were women. Women on average had five times the incidence and seven times the prevalence of men. The differences in SLE by gender may be attributed to differences in the metabolism of sex hormones and/or gonadotropin-releasing hormone (GnRH) signalling.^21^ This also led to the gender difference at age distribution of SLE patients: women had the highest incidence of SLE at the younger age groups while men had the highest incidence at the older age group. It was reported that during the childbearing age, the ratio of females to males is nine to one, with a lower ratio seen before puberty and a decline later in life.^22^ This is consistent with what has been found in this study showing that the ratio of SLE incidence for women to men was ten at the age group of 20–29 years, but only two at the age of 0–9 years and two at the age of 80 years or older.

Pacific people had the highest incidence and prevalence of SLE, with four times the incidence rate and three times the prevalence rate higher than Europeans/others. Māori and Asian also had around twice the incidence and prevalence of Europeans/others. The ethic differences in SLE may be associated with the natural history of disease. The cause of SLE is unknown, but it is believed to result from a complex interaction between genetics, environmental exposures and hormones.^23^ In New Zealand, Pacific people die younger and have higher rates of chronic diseases than other New Zealanders.^24^ Social and economic factors are known to contribute significantly to their relatively poorer health status.^24^ Over half of Māori and Pacific SLE patients were living in the most deprived areas compared to 18.0% Asian and 14.5% European/Others. Because of a lack of population data by deprivation, we could not estimate the incidence nor prevalence rate by deprivation. Regarding the impact of socioeconomic status on SLE incidence and prevalence, the results from overseas studies are contradicting. For example, a study in Taiwan found that for people with highest income group, the overall relative risk of SLE was lower.^18^ However, studies in the Swedish^25^ and Korea^26^ showed that unemployment, dismissal and income levels are not associated with the incidence of SLE.

This is a large, national study with a population of over 4 million people, across a 17-year period (2005–2021). The number of included SLE patients was bigger than any other existing New Zealand studies. The big number of patients also enabled us to examine the differences in incidence and prevalence by ethnic group. We have also validated the methods of patient identification using the Waikato data and only included the patients in 2010–2021 to improve the accuracy. However, this study has some limitations. The SLE patients were identified from NMDS and Mortality data, therefore SLE patients who were not in these two datasets were not identified. The incidence and prevalence may be underestimated. From the data validation using the Waikato patients, we found that around 90% of patients had a correct or near correct year of diagnosis, but there were still 10% of patients had a year of diagnosis that was more than 5 years away from the actual year of diagnosis. Therefore, the incidence by age group was probably underestimated in the younger age groups. However, these national administrative datasets are still valuable sources for research, produce useful information to guide practice and are commonly used for lupus research.^27–30^ The clinical notes in the Waikato Hospital did have the statement that the patients had SLE but did not specify what classification criteria for SLE the patients fulfilled. Patients diagnosed with SLE by a rheumatologist would likely fulfil the American College of Rheumatology (ACR) criteria^31^ which is predominantly used in New Zealand. However, it is still a limitation in this study. These criteria were developed to help standardise people for clinical trials and not for routine clinical practice which is where our data set comes from.

## Conclusions

The incidence and prevalence of SLE in New Zealand were comparable to the rates in European countries. The incidence of SLE in New Zealand has been stable over time, but the prevalence has been increasing, especially for Pacific people. Pacific people had the highest incidence and prevalence of SLE, more than three times the rates for Europeans/others. The high incidence of SLE in Māori and Asian people also has implications for the future as these populations increase as a proportion to the total population.

## Supplemental Material


Supplemental Material - Incidence and prevalence of systemic lupus erythematosus in New Zealand from the national administrative datasets
Supplemental Material for Incidence and prevalence of systemic lupus erythematosus in New Zealand from the national administrative datasets by Chunhuan Lao, Douglas White, Kannaiyan Rabindranath, Philippa Van Dantzig, Donna Foxall, Apo Aporosa, and Ross Lawrenson in Lupus
