# Supplementary material for: Incidence and prevalence of systemic lupus erythematosus in New Zealand from the national administrative datasets
Source: Lupus. 2023 Jun 2;32(8):1019–27. doi: 10.1177/09612033231182203 (PMC10333972; doi:10.1177/09612033231182203)
Supplement: Supplemental Material - Incidence and prevalence of systemic lupus erythematosus in New Zealand from the national administrative datasets [file sj-pdf-1-lup-10.1177_09612033231182203.pdf]

Appendix Table 1. Gap between the first date of SLE identification in the Waikato hospital records and the date from the national administrative datasets

Gap between the two dates	Year of first identification of SLE in the national administrative datasets				Total	
	2005-2009		2010-2021			
0 year	46	45.5%	69	78.4%	115	60.8%
1-5 years	25	24.8%	10	11.4%	35	18.5%
6-10 years	20	19.8%	3	3.4%	23	12.2%
>10 years	10	9.9%	6	6.8%	16	8.5%
No lupus information†	7		4		11	
Total	108		92		200	

† This is because these patients immigrated to the Waikato region recently, and the diagnosis of SLE was made in other regions.

Appendix Table 2. Age-standardised incidence of SLE and 95% confidence interval by ethnic group

Subgroup	2010	2011	2012	2013	2014	2015	2016	2017	2018	2019
**Women**										
All	5.0 (4.1-5.9)	4.0 (3.2-4.8)	4.2 (3.4-5.1)	3.2 (2.5-4.0)	3.5 (2.8-4.3)	3.7 (2.9-4.5)	4.0 (3.2-4.8)	3.0 (2.4-3.7)	2.4 (1.8-3.0)	3.2 (2.4-3.9
Māori	6.7 (3.7-9.6)	7.9 (4.7-11.1)	4.4 (2.0-6.7)	2.8 (0.9-4.7)	3.0 (1.0-4.9)	2.5 (0.8-4.2)	4.6 (2.3-6.9)	3.2 (1.4-5.0)	1.9 (0.5-3.4)	2.2 (0.8-3.6
Pacific	17.1 (10.0-24.3)	12.2 (6.4-17.9)	12.5 (6.6-18.4)	10.7 (5.3-16.2)	6.6 (2.5-10.7)	14.4 (8.5-20.2)	8.7 (4.3-13.2)	7.8 (3.6-12.1)	7.2 (3.5-11.0)	7.9 (4.1-11.
Asian	6.5 (3.3-9.7)	5.0 (2.2-7.9)	4.4 (1.8-6.9)	5.7 (2.8-8.6)	9.1 (5.7-12.5)	6.3 (3.5-9.1)	6.5 (3.8-9.2)	5.8 (3.4-8.3)	3.5 (1.6-5.4)	4.6 (2.5-6.7
Europeans /Others	3.0 (2.2-3.8)	2.2 (1.6-2.9)	3.2 (2.4-4.1)	2.2 (1.5-2.9)	2.1 (1.4-2.8)	2.3 (1.5-3.1)	2.4 (1.6-3.2)	1.5 (0.9-2.1)	1.5 (0.9-2.1)	1.9 (1.2-2.5
**Men**										
All	0.8 (0.4-1.1)	0.8 (0.4-1.1)	0.5 (0.2-0.8)	0.7 (0.3-1.0)	0.6 (0.3-0.9)	0.9 (0.5-1.2)	0.6 (0.3-0.9)	0.5 (0.3-0.8)	0.4 (0.2-0.7)	0.4 (0.2-0.6
Māori	1.2 (0.0-2.5)	1.1 (0.0-2.6)	1.0 (0.0-2.2)	0.8 (0.0-1.8)	0.9 (0.0-2.0)	0.4 (0.0-1.2)	0.3 (0.0-0.9)	0.3 (0.0-0.9)	0.3 (0.0-0.8)	1.2 (0.0-2.3
Pacific	1.8 (0.0-4.2)	3.3 (0.1-6.5)	0.0	0.8 (0.0-2.3)	3.7 (0.5-7.0)	1.0 (0.0-2.3)	1.0 (0.0-2.4)	1.2 (0.0-2.8)	1.9 (0.0-4.2)	0.0
Asian	0.9 (0.0-2.2)	1.4 (0.0-2.9)	0.9 (0.0-2.1)	0.5 (0.0-1.6)	1.7 (0.0-3.4)	1.6 (0.2-3.0)	0.8 (0.0-1.8)	0.4 (0.0-1.1)	0.5 (0.0-1.2)	0.3 (0.0-0.8
Europeans /Others	0.6 (0.2-0.9)	0.5 (0.2-0.5)	0.3 (0.1-0.5)	0.7 (0.3-1.1)	0.2 (0.0-0.5)	0.6 (0.3-1.0)	0.4 (0.1-0.7)	0.5 (0.2-0.9)	0.2 (0.0-0.4)	0.3 (0.1-0.6

Appendix Table 3. Age-standardised prevalence of SLE and 95% confidence interval by ethnic group

2011	2012	2013	2014	2015	2016	2017	2018	2019	2020	
61.9 (58.8-65.0)	63.7 (60.5-66.8)	64.6 (61.5-67.8)	65.5 (62.3-68.6)	66.1 (62.9-69.2)	67.2 (64.1-70.3)	67.2 (64.1-70.3)	66.5 (63.5-69.6)	66.7 (63.7-69.7)	66.3 (63.3-69.3)	6
89.1 (78.1-100.1)	91.5 (80.4-102.5)	90.9 (79.9-101.8)	89.0 (78.5-99.6)	86.3 (76.1-96.4)	85.0 (75.2-94.9)	83.3 (73.8-92.8)	79.6 (70.5-88.7)	76.7 (67.9-85.4)	75.8 (67.3-84.4)	7
170.3 (147.7-192.9)	176.8 (154.0-199.7)	184.1 (160.9-207.2)	180.2 (157.9-202.5)	181.9 (160.1-203.7)	180.8 (159.6-202.0)	179.0 (158.3-199.7)	177.1 (156.9-197.2)	176.7 (157.0-196.3)	173.2 (154.1-192.4)	
68.7 (58.7-78.7)	69.7 (59.9-79.6)	71.7 (61.9-81.6)	74.6 (64.9-84.2)	74.6 (65.3-83.9)	75.0 (66.0-84.0)	75.5 (66.8-84.2)	73.2 (64.9-81.6)	73.3 (65.1-81.5)	72.6 (64.7-80.6)	7
45.9 (42.9-49.0)	47.1 (44.0-50.2)	47.6 (44.4-50.7)	48.3 (45.1-51.4)	48.9 (45.8-52.1)	49.8 (46.6-53.0)	49.6 (46.4-52.8)	49.6 (46.5-52.8)	49.9 (46.7-53.0)	49.9 (46.8-53.1)	4
7.9 (6.8-9.1)	8.1 (6.9-9.2)	8.3 (7.2-9.5)	8.6 (7.4-9.7)	8.9 (7.7-10.0)	8.8 (7.7-10.0)	8.9 (7.8-10.1)	8.9 (7.8-10.0)	8.8 (7.7-9.9)	8.9 (7.8-10.0)	
12.0 (7.6-16.5)	11.8 (7.5-16.1)	11.5 (7.3-15.7)	11.6 (7.5-15.6)	11.2 (7.3-15.1)	10.9 (7.2-14.6)	10.6 (7.1-14.2)	10.0 (6.6-13.3)	10.4 (7.0-13.7)	11.2 (7.9-14.6)	1
22.8 (13.7-31.9)	22.2 (13.3-31.1)	21.6 (12.9-30.2)	23.9 (15.2-32.5)	23.8 (15.4-32.1)	23.4 (15.4-31.4)	21.1 (13.9-28.3)	22.1 (14.9-29.3)	19.8 (13.2-26.4)	19.6 (13.2-26.1)	1
10.4 (6.1-14.6)	10.7 (6.5-14.9)	10.9 (6.7-15.1)	11.6 (7.4-15.7)	12.4 (8.3-16.5)	12.2 (8.3-16.1)	11.7 (8.0-15.4)	11.5 (7.9-15.0)	11.0 (7.6-14.3)	11.2 (7.9-14.5)	1
5.9 (4.9-7.0)	5.9 (4.9-7.0)	6.4 (5.3-7.5)	6.3 (5.2-7.4)	6.5 (5.4-7.7)	6.5 (5.4-7.6)	6.8 (5.6-7.9)	6.7 (5.6-7.9)	6.7 (5.6-7.8)	6.7 (5.6-7.8)	
